# Blood–brain barrier and foetal-onset hydrocephalus, with a view on potential novel treatments beyond managing CSF flow

**DOI:** 10.1186/s12987-017-0067-0

**Published:** 2017-07-13

**Authors:** M. Guerra, J. L. Blázquez, E. M. Rodríguez

**Affiliations:** 1 Instituto de Anatomía, Histología y Patología, Facultad de Medicina, Universidad Austral de Chile, Valdivia, Chile; 2 Departamento de Anatomía e Histología Humana, Facultad de Medicina, Universidad de Salamanca, Salamanca, Spain

**Keywords:** Foetal-onset hydrocephalus, Blood–brain barrier, Cerebrospinal fluid, Cell therapy

## Abstract

Despite decades of research, no compelling non-surgical therapies have been developed for foetal hydrocephalus. So far, most efforts have pointed to repairing disturbances in the cerebrospinal fluid (CSF) flow and to avoid further brain damage. There are no reports trying to prevent or diminish abnormalities in brain development which are inseparably associated with hydrocephalus. A key problem in the treatment of hydrocephalus is the blood–brain barrier that restricts the access to the brain for therapeutic compounds or systemically grafted cells. Recent investigations have started to open an avenue for the development of a cell therapy for foetal-onset hydrocephalus. Potential cells to be used for brain grafting include: (1) pluripotential neural stem cells; (2) mesenchymal stem cells; (3) genetically-engineered stem cells; (4) choroid plexus cells and (5) subcommissural organ cells. Expected outcomes are a proper microenvironment for the embryonic neurogenic niche and, consequent normal brain development.

## Background

Foetal-onset hydrocephalus is a heterogeneous condition. Genetic [1] and environmental factors, such as vitamin B or folic acid deficiency [2], viral infection of ependyma [3], and prematurity-related germinal matrix and intraventricular hemorrhage [4], contribute to its occurrence. Recent studies have begun to identify the cellular pathologies that accompany foetal-onset hydrocephalus. Studies on numerous mutant animal models indicate that a disruption of the ventricular zone (VZ) of the cerebral aqueduct, starting early in development, triggers aqueduct stenosis and hydrocephalus [5–7]. A similar phenomenon seems to take place in cases of human foetal-onset hydrocephalus [8, 9]. The process of VZ disruption, which first affects the cerebral aqueduct, but also reaches the telencephalon, results in two neuropathological events: the formation of subependymal grey matter heterotopia (also known as ‘periventricular heterotopia’), resulting from a failure of neuroblast migration during development of the embryonic brain, and the translocation of neural stem cells/neural progenitor cells into the foetal cerebrospinal fluid (CSF) [7, 10, 11]. Cerebral abnormalities are irreversible inborn defects and they could explain some of the neurologic impairments (e.g. epilepsy) of children born with hydrocephalus.

**Fig. 1:**
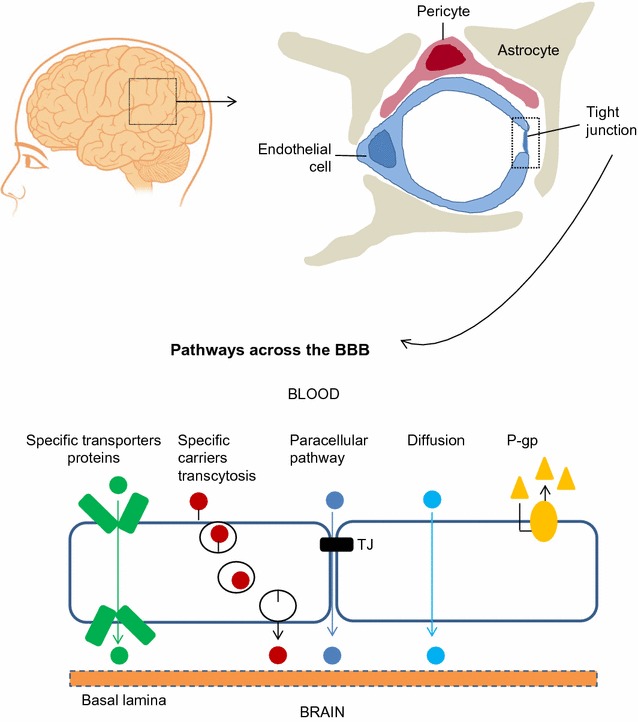
Cellular constituents of the blood–brain barrier. The blood–brain barrier is formed by brain endothelial cells, which are connected by tight junctions. The endothelium, together with the basal lamina, pericytes, and astrocytic end-feet forms the neurovascular unit. Transport pathways across blood brain barrier. Endothelial cells of the BBB have a crucial role in the transport of ions and solutes into and out of the brain. Some substances diffuse freely into and out of the brain parenchyma (O_2_ and CO_2_), others such as nutrients need specific transporters, while molecules such as insulin, leptin and transferrin are transported by receptor-mediated transcytosis. *P-gp* P-glycoprotein, *TJ* tight junction

Foetal-onset hydrocephalus affects 1–3 of 1000 live births and is characterized by abnormal CSF flow accompanied by ventricular dilatation [12]. Although surgical diversion of CSF with shunts does prevent further damage to the brain caused by hydrocephalus, it does not solve the essential brain maldevelopment and neurological outcome associated with hydrocephalus. Indeed, 80–90% of the neurologic impairment suffered by shunt-dependent neonates with foetal-onset hydrocephalus is not reversed by surgery [13, 14]. The treatment of neurologic disorders is challenging because of the brain barriers that make it difficult to effectively and persistently deliver therapeutic compounds. The tight endothelial barrier can be bypassed using endogenous blood–brain barrier (BBB) transporters allowing carrier-mediated transport or receptor-mediated transport [15–17] (Fig. 1).

Recent years have witnessed research progress in the development of cell therapies for brain diseases, including neurological impairment associated with the onset of hydrocephalus. Expected applications for cell therapy are the regeneration of the disrupted VZ and drug delivery to improve the brain microenvironment and neurological function. We discuss this evidence in the present review.

## Ontogenetic development of the blood–brain barrier (BBB) in animals and humans

The idea of a blood–brain barrier (BBB) that segregates blood from brain was developed 100 years ago, following the demonstration that vital dyes injected intravenously stained most organs but not the brain and spinal cord [18, 19]. The spatial organization of the barrier is complex, and although at its various locations (brain parenchyma, meninges, choroid plexus) it is formed by different cell types (endothelium, mesenchymal cells of meninges, choroidal cells), it behaves as a single, tight and fully efficient barrier [20, 21]. Adding further levels of complexity, there are discrete brain areas, known as circumventricular organs, in which the BBB is displaced from the endothelial site to the ependymal side, allowing small regions of the CNS to be directly exposed to blood without making the BBB generally leaky [20, 21].

The different cell organization of the barrier at its various brain locations allows it to display distinct barrier and permeability properties. Such innate barriers are dynamic and complex interfaces that strictly control the exchange between blood or CSF and brain compartments. Major barrier functions include: (1) maintenance of CNS homeostasis; (2) protection of the private neural environment from that of the blood; (3) provision of a constant supply of nutrients to the brain; (4) To convey inflammatory cells to specific sites in response to changes in the local environment [22, 23]. Several cell types contribute to the organization of the BBB, also known as the neurovascular unit, located at the capillaries in the brain parenchyma. Endothelial cells are at the heart of the BBB; pericytes control the expression of specific genes in endothelial cells; astrocytes convey molecules from and to the tight endothelium and contribute to the maintenance of the barrier postnatally [24–26]. Further, recent evidence has highlighted the role of neural activity in promoting the maturation of cerebrovascular networks during postnatal development [27].

The polarized nature of CNS endothelial cells is reflected in their four fundamental barrier properties that contribute to BBB function and integrity. First, tight junction (TJ) complexes between endothelial cells establish a high-resistance paracellular barrier to small hydrophilic molecules and ions. Second, in endothelial cells the transcellular vesicular trafficking of cargo molecules is limited to the receptor-mediated endocytosis/transcytosis. Third, the establishment of the restrictive paracellular and transcellular barriers allows CNS endothelial cells to use polarized cellular transporters to dynamically regulate the influx of nutrients and efflux of metabolic waste and toxins between the blood and brain parenchyma. Fourth, CNS endothelial cells lack the expression of leukocyte adhesion molecules (LAMs) such as E-selectin and Icam. The lack of these luminal surface molecules prevents the entry of immune cells from blood, resulting in a paucity of immune cells in the brain microenvironment [16]. BBB properties are not intrinsic to CNS endothelial cells but are induced and regulated by the neural environment [28].

The development of the BBB is a multistep process that begins with angiogenesis [29]. Barrier properties mature as nascent vessels come into close contact with pericytes and astroglia. This process includes elaboration of TJ, decreased transcytosis, downregulation of leukocyte adhesion molecules and increased transporter expression [30–33]. Full tightness of TJ is completed during maturation and needs to be maintained throughout life. If the barrier breaks down, there can be dramatic consequences, and neuroinflammation and neurodegeneration can occur [33–35]. Recently, neurovascular dysfunction, including BBB breakdown and cerebral blood flow dysregulation and reduction, has been recognized to contribute to Alzheimer’s disease [35] and epilepsy [36].

The temporal profile of BBB development varies with species. In addition to tracer injections, the ultrastructure cellular properties of endothelial cells, the onset of specific BBB marker expression, and the presence of endogenous serum proteins in brain parenchyma have been used to study how barrier properties develop.

In humans, the vascularisation of the telencephalon begins at approximately the 8th week of gestation (GW). Post-mortem studies of preterm foetuses have shown that a barrier to trypan blue is present at the beginning of the second trimester of gestation [37]. By the 14th GW TJ proteins occludin and claudin-5 are expressed in the vessels of the germinal matrix, cortex and white matter [38]. The appearance of TJ proteins at this time appears sufficient to prevent endogenous albumin from entering the brain, providing evidence of early functionality of the barrier [38]. By the 18th week of gestation, TJ proteins demonstrate similar staining patterns to the TJ of the adult BBB [39]. Recruitment of pericytes to the developing capillary wall is critical for the formation and maintenance of the BBB. Astrocytes recruited at later stages further assist endothelium in acquiring BBB characteristics, barrier properties and CNS immune quiescence [23–26].

Some pathways have been implicated in the pericyte-mediated induction and regulation of the BBB. The best characterized genetic program is β-catenin signalling [40–42]. CNS-specific pathways (Wnt/β-catenin, Norrin/Frizzled4 and sonic hedgehog) [43] and genes (GPR124, Mfsd2a, apoE3) are also crucial in BBB differentiation and maturation [44, 45]. Loss-of-function of these genes results in CNS vasculature dysfunction.

In brief, methodological and technical achievements have allowed to establish that humans, rodents, and other animals (i.e. sheep, rabbits, chicken) [46–51] have a number of functional barrier mechanisms in place early in development. These include TJ proteins and several transporters. BBB develops in a caudal-rostral wave with the hindbrain BBB becoming functional first followed by the midbrain, and finally the forebrain [44]. Barrier transporting properties are induced very early. In contrast, barrier sealing properties are acquired gradually throughout development, first with the suppression of fenestrations, then the appearance of functional TJ and lastly with the suppression of transcytosis [30–33]. These findings are controversial because they support the view of a functional embryonic BBB protecting the developing brain and oppose the traditional perspective that “the vulnerable developing brain is only protected by the barrier properties of the placenta” [52] [for more comprehensive reviews see 53, 54].

The progressive maturation of the BBB components (i.e., expression of TJ proteins) should not be interpreted as a fully functionally operative barrier. When in development (pre- or postnatal) does the BBB starts to operate as a true, unique and fully tight barrier? This a key question from the physiological, pathological and therapeutic points of view. A functional BBB during the embryonic life implies that the nervous system develops in a defined and restricted environment; is this really the case? Does a functional embryonic BBB protect the embryonic brain from compounds (toxins or drugs) that escape the placental barrier? How does BBB dysfunction during embryogenesis impact brain development? Although BBB dysfunction has been associated with the initiation and persistence of various neurological disorders in the adult [33–36] and developing brain [55], it is unclear whether barrier dysfunction is a cause or a consequence of a particular neurological disorder. This is an area in which further research with modern technology is required [56]. On the other hand, a truly and fully functional BBB represents a challenge when targeting treatments towards the mother or foetus, or in the treatment of premature newborns.

## Abnormalities of BBB in foetal-onset hydrocephalus: trigger or outcome?

All cells of the mammalian central nervous system are produced in two germinal zones associated with the ventricular walls, the ventricular zone (VZ) and the subventricular zone (SVZ) [57]. In the human, the SVZ has a massively expanded outer region that contributes to the large size and complexity of the brain cortex [58–61]. Although the bulk of neural proliferation and neuroblast migration occurs between 12 and 18 GW, it continues at a decreasing rate until about the 34th GW. As neurogenesis declines, ependymogenesis takes over (20–40 GW) by the progressive differentiation of neural stem cells (NSC) into multiciliated ependyma [57].

Over the years, based on our own and other investigators’ evidence, we have progressively come to the view that a disruption of the VZ and SVZ, affecting equally NSC and ependymal cells, triggers the onset of foetal hydrocephalus and abnormal neurogenesis [7, 10, 11; Fig. 3a]. Disruption follows a program that has a temporal and spatial pattern, progressing as a “tsunami” wave running from the caudal to rostral regions of the developing ventricular system, leaving behind severe damage. In the hyh mice and HTx rat, animal models of foetal-onset hydrocephalus, the onset of VZ disruption is associated with the arrival of macrophages and lymphocytes to the zone that has just started to denude [6, 62], suggesting that an inflammatory/immune response could be associated with the progression and severity of hydrocephalus. Supporting this view, in the hyh mouse, the tumour necrosis factor alpha (TNFα) and its receptor TNFαR1 appear to be associated with the severity of the disease [63]. In human neonatal high pressure hydrocephalus, pro-inflammatory cytokines (IL-18 and IFNgamma) have been detected in the CSF [64].

At present, there is little information whether or not the BBB is affected in hydrocephalus. Recent studies have shown that at the neurovascular unit, endothelial cells, astrocytes, and pericytes synthesise and deposit different laminin isoforms into the basal lamina. Laminin α4 (endothelial laminin) regulates vascular integrity at embryonic/neonatal stage, while astrocyte laminin maintains vascular integrity in adulthood [65, 66]. The loss of pericyte laminin leads to hydrocephalus and BBB breakdown [67]. At variance, in the capillaries of the hydrocephalic HTx rat laminin immunoreactivity at the BBB is not different from that of control rats [68]. In HTx rats, tight junctions between endothelial cells of capillaries are apparently well formed and capillaries with partial defect of the basal membrane are occasionally found. However, the swelling of astrocytic end-feet around microvessels located in areas of injured white matter was interpreted as impairment of the BBB [68].

Other studies have focused on the role of aquaporins in the pathophysiology of hydrocephalus. Aquaporin-1 is highly expressed at the choroid plexus and is related to CSF production; aquaporin-4 is expressed at the ependyma, glia limitans, and at the perivascular end feet of astrocyte processes, facilitating the water movement across these tissue interfaces [69–71]. So far, the observations obtained from animal studies [72–75] and few cases in humans [74, 76] support an adaptive and protective role of aquaporins in hydrocephalus by decreasing CSF production and increasing edema clearance [77].

Although the evidence is poor, the possibility that an inflammatory process is somehow associated with the early stages of VZ disruption deserves to be explored. Pro-inflammatory interleukins have been detected in the CSF of hydrocephalic mutant rodents [63, 78], hydrocephalic patients [64, 76, 79]. It is well known that neuroinflammation is generally accompanied by impaired BBB function, which includes alterations in the junctional complexes [80–83]. Vascular endothelial growth factor (VEGF), which expression is significantly up-regulated during neuroinflammation, induces disruption of BBB, likely by down-regulating claudin-5 and occludin [84, 85]. Interestingly, VEGF is elevated in the CSF of patients with hydrocephalus and, when it is administered into the CSF of normal rats, it causes alterations of adherens junctions (AJ), ependyma disruption, and hydrocephalus [86]. Stable AJ are now considered to be required for the formation of TJ [87]. Surprisingly, the continuous crosstalk between components of AJ and TJ has been underestimated by researchers studying the BBB and hydrocephalus. The possibility that signals from the hydrocephalic CSF (cytokines, VEGF, others) may contribute to, or even trigger, the BBB disruption should be kept in mind.

### Germinal matrix hemorrhage and the BBB

Germinal matrix (GM) haemorrhage and intraventricular haemorrhage (IVH) are the most common and most important events that cause neurological impairment in neonates born before 37 GW [88]. IVH occurs when a hemorrhage in the germinal matrix ruptures through the ependyma into the lateral ventricles, leading to hydrocephalus and other long-term sequelae. Prematurity associated with posthaemorrhagic hydrocephalus (PHH) results in high morbidity and mortality. Infants with a history of IVH/PHH have a higher incidence of seizures, neurodevelopmental delay, cerebral palsy, and death [88–90]. The pathogenesis of IVH is multifactorial and it has been primarily ascribed to a combination of intravascular, vascular, and extravascular factors, including: (1) disturbance in the cerebral blood flow; (2) inherent fragility of the GM-vasculature; (3) platelet and coagulation disorders [for comprehensive reviews see 91, 92]. It has been suggested that all or some of these conditions could lead to significant fluctuation in the cerebral blood flow or blood pressure inside the blood vessels, and may participate in the rupture of the microvasculature [93].

**Fig. 2:**
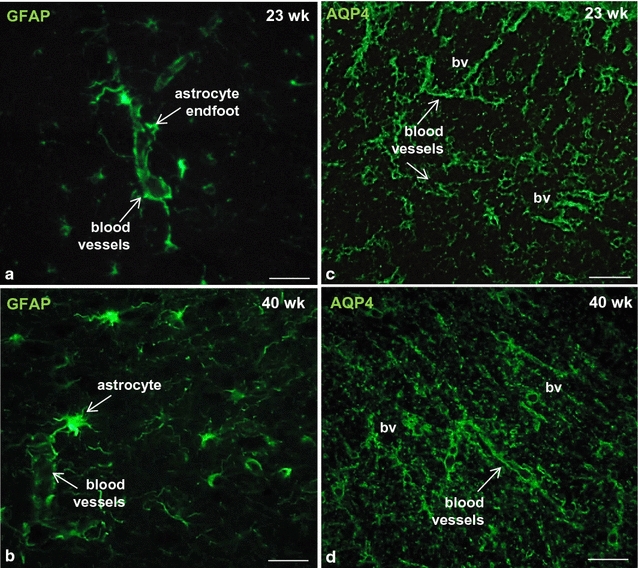
Blood brain barrier in the developing human cerebral cortex. Telencephalon of premature newborns. Immunostaining shows the presence of GFAP (**a**, **b**) and aquaporin-4 (**c**, **d**) around brain microvessels as early as 23 weeks of gestation. *bv* blood vessels. *Scale bars* **a**, **b** 10 µm; **c**, **d** 30 µm

The morphology and functional properties of the GM-gliovascular interface have been studied in human embryos. The perivascular coverage by the end-feet of GFAP-reactive astrocytes increases consistently from 19 to 40 GW [94]. In a similar way, tight junction length, basal lamina area in the GM-vasculature and aquaporin-4 expression in astrocyte end-feet increase as a function of gestational age [94–97] (Fig. 2). It is worth ning that a lower degree of GFAP expression in astrocyte end-feet of the GM vasculature, as compared to that of the developing cortex and white matter, has been reported. It has been suggested that it may reflect cytoskeletal structural differences that would contribute to the fragility of the GM-vasculature and susceptibility to hemorrhage [94]. In addition, poorly developed TJs between endothelial cells, or immaturity of the basal lamina and/or pericytes have been also suggested as a risk factor for IVH [37, 94, 97].

## Difficulties in the non-surgical treatment of hydrocephalus

Del Bigio and Di Curzio have recently written a critical review to summarize and evaluate research concerning pharmacological therapies for hydrocephalus [98]. Some approaches currently used to deliver therapeutic compounds to the brain include transcranial drug delivery, transnasal drug delivery, transient BBB opening, and small molecule lipidization [99–101]. Delivery of therapeutic compounds into the CSF is also emerging as an alternative. What has been the aim, so far, of all surgical and pharmacological attempts to treat hydrocephalus? (1) To repair disturbances in CSF flow/balance and (2) to prevent brain *damage* caused by hydrocephalus. Despite over five decades of research, no compelling non-derivative therapies have been developed for hydrocephalus. An alternative and promising task has recently been proposed: to prevent or diminish *abnormalities in brain development* which are inseparably associated with hydrocephalus.

## Is there a real possibility to prevent/diminish brain abnormalities linked to foetal-onset hydrocephalus?

We believe that there is a hope. New medical technology could change the way to treat hydrocephalus and its outcomes, as a complement to CSF diversion by shunt surgery. Cell grafting therapy for brain diseases has been the subject of numerous publications. A few recent investigations have started to set the basis for a cell therapy for foetal-onset hydrocephalus. Potential cells to be used for brain grafting include: (1) pluripotential neural stem cells; (2) mesenchymal stem cells; (3) genetically engineered stem cells; (4) choroid plexus cells and (5) subcommissural organ cells. Expected outcomes are a proper microenvironment of the embryonic neurogenic niche and, consequently, normal brain development.

### Neural stem cells

**Fig. 3:**
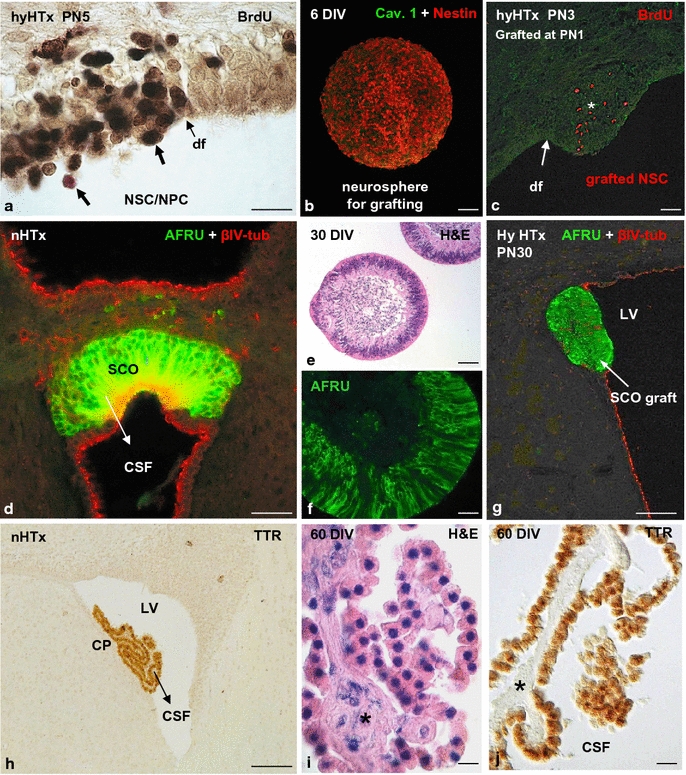
**a** In the hydrocephalic HTx rat at postnatal day 5, ventricular zone disruption results in abnormal translocation of neural stem cells (NSC)/neural precursor cells (NPC) into ventricular cerebrospinal fluid. Cells displaced to the ventricle and reaching the CSF retain proliferative capacity, as shown by injection of bromodeoxyuridine (BrdU) in living animals and tracking the BrdU-positive cells in tissue sections (*arrows*). *df* disruption front. **b**, **c** Neural stem cells (NSC) grafted into the cerebrospinal fluid (CSF) of a hydrocephalic HTx rat move selectively to the disrupted areas of the ventricular zone (VZ). Dispersed cells were grown in a neurosphere culture medium containing epidermal growth factor (EGF) and devoid of fetal bovine serum. Neurosphere immunostained for nestin after 6 days in vitro (DIV, **b**). Some of the grafted cells migrate through the subventricular zone; some of them move deeper into the brain tissue (*asterisk*, **c**). **d** Frontal section of the rat subcommissural organ (SCO) immunostained with antibodies against SCO-spondin (AFRU) and βIV-tubulin. *CSF* cerebrospinal fluid. **e**, **f** Organ culture of the bovine SCO. **e** After 30 DIV, SCO explants form spheres of secretory ependymocytes. Section of an SCO-explant stained with haematoxylin-eosin. **f** Section of a SCO-explant immunostained with AFRU. **g** Bovine SCO explant grafted into the lateral ventricle of a hydrocephalic HTx rat. The graft becomes integrated into the wall of the lateral ventricle (LV). SCO-spondin immunoreactive material is shown inside the cells. **h** Frontal section of a rat brain immunostained with antibodies against transthyretin (TTR).The choroid plexus (CP) is selectively immunoreactive. **i**, **j** Organ culture of the bovine choroid plexus. **i** Section of a CP-explant stained with haematoxylin-eosin. **j** Section of a CP-explant immunostained with anti-transthyretin. After 60 DIV, the choroid cells display a normal cytology and continue to express TTR. The vasculature and stroma of the villi were virtually missing (*asterisk*). *Scale bars* **a** 15 µm; **b**–**g** 50 µm; **h** 100 µm; **i**, **j** 12 µm. **a**–**c** were taken from Rodriguez et al. [11]. *Reprinted with permission of Pediatr Neurosurg*; **d** was taken from Ortloff et al. [151]. *Reprinted with permission of Cell Tissue Res*; **e**, **g** were taken from Guerra et al. [10]. *Reprinted with permission of JNEN*

Based on the evidence that the common history of foetal-onset hydrocephalus and abnormal neurogenesis starts with the disruption of the VZ, neurospheres formed by normal neural stem cells/neural progenitor cells (NSC/NPC) have been grafted into the lateral ventricle of hydrocephalic HTx rats for regenerative purposes (for comprehensive reviews see 11, 102). After 48 h of transplantation, the grafted cells become selectively integrated into the areas of VZ disruption [11]; Fig. 3b, c. Although the further fate of these cells is under investigation in our laboratory, the possibility to repopulate the disrupted VZ with neural stem cells (radial glia) and ependymal cells, avoiding the outcomes of VZ disruption (hydrocephalus and abnormal neurogenesis), may be in sight. Recently, the combination of endogenous NSC mobilization and lithium chloride treatment resulted in highly reduced incidence of hydrocephalus by inhibiting neuronal apoptosis in a rodent model of intraventricular haemorrhage [103].

The isolation and expansion of NSC of human origin are crucial for the successful development of cell therapy approaches in human brain diseases. A relevant step forward has been recently achieved in that an immortal foetal neural stem cell line [104] and a foetal striatum-derived neural stem cell line [105] has been obtained.

### Mesenchymal stem cells

Mesenchymal stem cells (MSC) are versatile and multipotent adult stem cells. MSC are capable of differentiating into osteoblasts, chondroblasts, myocytes, and adipocytes [106, 107]. Furthermore, neuronal progenitor cells, as well as lung epithelial and renal tubular cells, can be derived from MSC [108]. MSC represent an alternative source of stem cells that can be harvested at low cost and isolated with minimal invasiveness. There are large MSC populations in umbilical cord blood, placental membranes and amniotic fluid [109, 110]. MSC are emerging as a replacement for NSC for therapeutic purposes, specifically for their plasticity, their reduced immunogenicity, and high anti-inflammatory potential [111]. It is now becoming clearer that they might be able to protect the nervous system through mechanisms other than cell replacement, such as the modulation of the immune system [111] and the release of neurotrophic factors [112, 113].

Mesenchymal stem cells have been used for the treatment of posthemorrhagic hydrocephalus. The intraventricular transplantation of MSC in an intraventricular haemorrhage model of newborn rats significantly attenuated inflammatory cytokines of the cerebrospinal fluid and brain tissue, and prevented the development of posthemorrhagic hydrocephalus [114]. The mechanism of protection seems to be related to the anti-inflammatory effects of these cells and the capacity of MSC to release the brain-derived neurotrophic factor [112, 113]. Substantial evidence has been obtained for the successful treatment of brain diseases, such as Parkinson’s, using brain grafting of stem cells of various sources [115–117].

In brief, all these findings support that stem cells are promising therapeutic agents for brain regeneration and neuroprotection. A key point to consider is the time and opportunity when NSC should be transplanted. In normal human foetuses, neuronal proliferation and migration occur from the 12th to 30th GW, while in hydrocephalic foetuses VZ disruption starts at about the 16th GW and continues throughout the 2nd and 3rd trimester of pregnancy [118]. It seems reasonable to suggest that NSC grafting should be performed shortly after the disruption process of the VZ had been turned on. Foetal surgery to repair neural tube defects, such as spina bifida, is performed within a well-defined gestational period (19th–25th GW) according to the MOMS study [119]. This operation, that is becoming progressively standardized and safe, appears to be a good opportunity for NSC grafting into the brain ventricles of spina bifida foetuses. Worth mentioning is the fact that foetuses with spina bifida carry a VZ disruption [9, 10] and most children born with spina bifida have hydrocephalus. It may be hoped that grafting of stem cells into brain of hydrocephalic foetuses would result in the repopulation of the disrupted areas of the VZ and/or the generation of a protective microenvironment to diminish/prevent the outcomes of VZ disruption, namely, hydrocephalus and abnormal neurogenesis.

Warnings about unwanted outcomes of stem cell transplantation should be kept in mind permanently. The existing evidence supports that the short term application of stem cells is safe and feasible; however, concerns remain over the possibility of unwanted long-term effects [120–122]. In addition to unwelcome interactions of stem cells with the host immune system, there is evidence that they may promote tumorogenesis [123]. As animal models and first-in-man clinical studies have provided conflicting results, it is challenging to estimate the long-term risk for individual patients [124, 125]. Previous evidence has shown that the safety of stem cell therapies will depend on various factors including the differentiation status and proliferative capacity of the grafted cells, the timing and route of administration, and the long-term survival of the graft [126–130].

Human MSC have been also genetically engineered to release neuropeptides with neuroprotective potential such as brain-derived neurotrophic factor (BDNF), glial cell line-derived neurotrophic factor (GDNF) or insulin-like growth factor 1 (IGF-1) [131]. Glage et al. [132] grafted human MSC transfected to produce glucagon-like peptide-1 in the CSF of cats. This study showed that ventricular cell-based delivery of soluble factors has the capability to achieve concentrations in the CSF which may become pharmacologically active. Thus, genetically engineered stem cells should be also considered to deliver specific neuroprotective compounds to the central nervous system [131]. Despite the controversy about the pharmacokinetic limitations and the technical difficulties of ventricular drug delivery, the CSF pathway is a promising route of administration for soluble, highly biologically-effective neuropeptides [133, 134].

There are two brain glands, the choroid plexus (CP) and the subcommissural organ (SCO), that secrete proteins and peptides into the CSF, some of them with neurogenic and neuroprotective properties. These two glands play a key role in the secretion and flow of CSF, and participate in the physiopathology of hydrocephalus [135–138].

### Choroid plexus

The choroid plexus (CP) cells are the main source of CSF, providing a full complement of proteins, peptides, nucleosides and growth factors such as the basic fibroblast growth factor (bFGF), insulin growth factor (IGF-II), nerve growth factor (NGF), and transforming growth factor (TGF), which influence a multitude of brain functions, including neurogenesis, neuroprotection, neurite extension as well as neuronal survival in vitro and in vivo [135, 139]. The marker secretory protein of the CP is transthyretin (Fig. 3h), a carrier of thyroxin throughout the CSF [140]. The transthyretin/thyroxin complex has a relevant role in neuronal differentiation and synaptogenesis in particular [140–142]. Thus, choroid plexus through its secretion into the CSF regulates nervous system structure and function [136, 142].

Grafting of CP has been explored for therapeutic purpose in some neurodegenerative disorders [for comprehensive reviews see 143, 144]. Surprisingly, CP grafting has not yet been considered in the treatment of hydrocephalus. The long-term survival of organ cultured CP (at least 2 months; ongoing experiments in our laboratory) and transplanted CP cells in vivo [145–147] provide a sound base to explore such a strategy. Worth noticing is that organ-cultured CP do not secrete CSF but they do secrete neurotrophic factors, such as transthyretin (ongoing experiments in our laboratory) (Fig. 3i, j).

### Subcommissural organ

The subcommissural organ (SCO) is a distinctive ependymal secretory gland located at the entrance of the cerebral aqueduct. The SCO differentiates very early in ontogeny and remains fully active during the entire life span, secreting SCO-spondin to the CSF where it either assembles to form Reissner’s fiber (RF) or remains soluble and circulates throughout the CSF compartments [148, 149]. The RF, extending through the Sylvius aqueduct (SA), fourth ventricle and central canal of the spinal cord, is indispensable for maintaining the patency of the SA and the normal flow of CSF [150–152]. An inborn defect of the SCO results in hydrocephalus [137, 138, 152].

In addition to SCO-spondin, the SCO secretes transthyretin, FGF, and the S100β protein, which support embryonic brain development [153, 154]. We have recently provided evidence to propose that these factors have similar roles in adult neurogenesis, regulating proliferation, migration and differentiation of neural stem cells and neural precursors in adult neurogenic niches [149].

The long-term survival of CP (Fig. 3h–j) and SCO explants (Fig. 3d–g) when they are cultured or transplanted into the ventricular CSF [146, 155, 156] provide a sound base to explore a CP/SCO cell-based therapy. When transplanted in the CSF, CP and SCO explants would allow a constant source and a homogenous distribution of neurotrophic and neuroprotective proteins, facilitating a uniform exposure of these compounds to the brain cells.

In order to translate cell therapies to humans, two strategies are envisaged. (1) To graft cells of human origin, mainly of human foetuses and (2) to graft cells of non-human origin. In such a case, a key question has to be solved: how to avoid the host versus graft immune reaction when the source of transplanted tissue is a non-human species.

## Microencapsulation permits use of allo- and xeno-grafting without immunosuppression

**Fig. 4:**
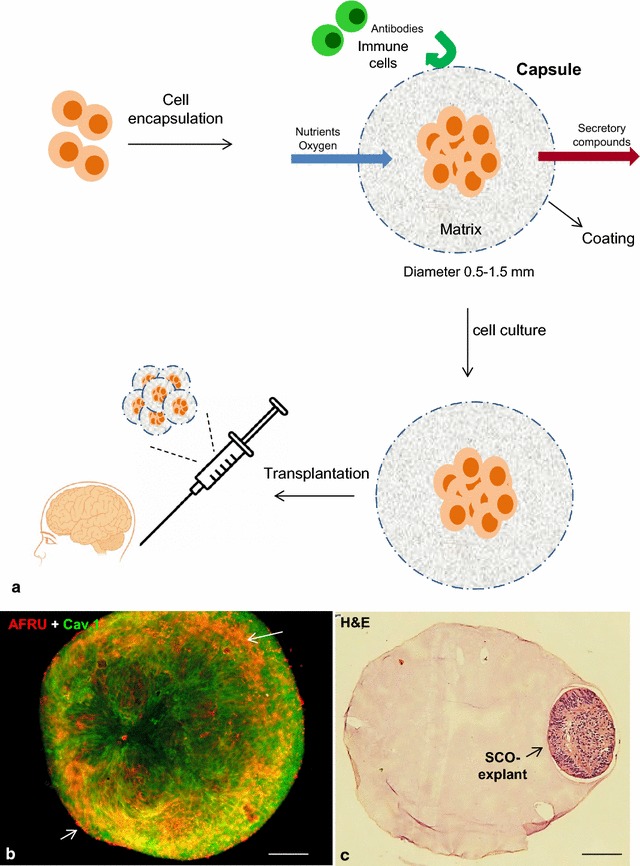
**a** Therapeutic application of encapsulated cells. Encapsulated cells are protected by a membrane or capsular matrix that allows nutrients, waste, and therapeutic products to pass freely but also works as a barrier to immune cells. Capsules can be transplanted into the brain for the treatment of brain diseases. **b** Subcommissural organ (SCO) secretory explants stained in bloc with AFRU (*red*) and caveolin-1 (*green*) displaying extracellular material (*arrows*) on the surface of cells. **c** Secretory SCO-explants encapsulated within a microsphere. Haematoxylin and eosin stain. *Scale bars* **b** 30 µm; **c** 160 µm

Cell encapsulation technology represents an alternative approach to the delivery of biologically active compounds to the brain by overcoming the problem of graft rejection [157]. This strategy involves the use of untreated or genetically engineered cells that secrete proteins with therapeutic potential. Cells are immobilized within a polymeric semi-permeable membrane that permits the bidirectional diffusion of molecules such as the influx of oxygen, nutrients, growth factors, essentials for cell metabolism and the outward diffusion of waste products and therapeutic proteins. At the same time, the semi-permeable nature of the membrane prevents the grafted cells being exposed to host immune cells and antibodies, avoiding their destruction (Fig. 4) [158, 159]. Through the release of therapeutic proteins, the grafted encapsulated cells can modify a circumscribed brain microenvironment or the whole brain milieu when transplanted into the CSF, and provide clinical benefits [132, 160, 161].

The use of an appropriate material with the property of biocompatibility is a crucial factor that governs the long term efficiency of this technology. The ideal capsule should need to be implanted only once in a patient’s lifetime; provide stable, predictable and reproducible function for a given period of time, and not burden the patient with immune suppressive regimens, discomfort, or other adverse effects. At present, alginates are regarded as the most suitable biomaterials for cell encapsulation due to their abundance and excellent biocompatibility properties [162, 163]. New polymers are being tested to be used as carriers and scaffolds for biomolecules and cell delivery in tissue engineering applications [159]. Encapsulation devices range from ‘microscale’ devices (100 nm–1 mm) to ‘macroscale’ (3–8 cm). Microcapsules, by virtue of their size, have a shorter diffusion distance for oxygen and other nutrients. However, they are mechanically and chemically fragile and cannot be retrieved once implanted within the brain parenchyma. Macrocapsules provide good cell viability and neurochemical diffusion, have good mechanical stability, and can be retrieved if needed or desired [159, 161].

Recent advances have increased the list of encapsulated cells that survive for a long-term in the brain and release therapeutic molecules ([161], Table 1); such neurotrophic factors do not cross the BBB.

**Table 1:** Examples of cell encapsulation for CNS and CNS-related diseases

Disease/model	Cells/experimental paradigm	Results and references
Alzheimer’s disease	NGF in rats and primates	Neuroprotection [164]
Parkinson’s disease	Neuropeptide Y in rats	Neuroprotection [165]
Huntington’s disease	Choroid plexus in rats	Neuroprotection [143]
Epilepsy	GDNF, BDNF in rats	Neuroprotection, Decreased seizures [166, 167]
Stroke/ischaemia	Choroid plexus in rats	Neuroprotection [168]
Acute and chronic pain	Chromaffin cells (catecholamines, opioids) in rats	Reduced pain [169]

## Conclusions

Although many agents have therapeutic potential for hydrocephalus, few of these agents have been clinically used because of the brain barriers. Virtually there are no reports trying to prevent or diminish abnormalities in brain development which are inseparably associated with hydrocephalus. Cell therapies for brain diseases, by grating cells with regenerative properties (stem cells) or able to secrete therapeutic compounds for an efficient period of time when they are transplanted into the CSF (MSC, CP, SCO), should be strongly considered for developing new treatments for hydrocephalus. The development in new technologies, such as cell encapsulation, will allow the use of foreign cells for transplantation, overcoming the existing problem of xenografts. A carefully considered decision process is indispensable before cell grafting in order to avoid unwanted results. Detailed observation and follow-up of the graft hosts should be a key compromise. To achieve the stem cells transplantation goal for hydrocephalus/spina bifida patients will require a balanced and complementary basic-clinical working team.

## Abbreviations

BBB: blood brain barrier
CP: choroid plexus
CSF: cerebrospinal fluid
GM: germinal matrix
IVH: intraventricular haemorrhage
MSC: mesenchymal stem cells
NPC: neural progenitor cells
NSC: neural stem cells
SCO: subcommissural organ
TJ: tight junction
VZ: ventricular zone
